# Tight junction disruption by cadmium in an in vitro human airway tissue model

**DOI:** 10.1186/s12931-015-0191-9

**Published:** 2015-02-21

**Authors:** Xuefei Cao, Haixia Lin, Levan Muskhelishvili, John Latendresse, Patricia Richter, Robert H Heflich

**Affiliations:** U.S. Food and Drug Administration/National Center for Toxicological Research, Jefferson, AR 72205 USA; Toxicologic Pathology Associates, Jefferson, AR 72079 USA; U.S. Center for Disease Control and Prevention, Atlanta, GA 30333 USA; Division of Genetic and Molecular Toxicology, 3900 NCTR Rd, Jefferson, AR 72079 USA

**Keywords:** Cadmium, Airway air-liquid-interface (ALI) culture, Tight junction, Occludin phosphorylation, Intracellular junctional interacting proteins

## Abstract

**Background:**

The cadmium (Cd) present in air pollutants and cigarette smoke has the potential of causing multiple adverse health outcomes involving damage to pulmonary and cardiovascular tissue. Injury to pulmonary epithelium may include alterations in tight junction (TJ) integrity, resulting in impaired epithelial barrier function and enhanced penetration of chemicals and biomolecules. Herein, we investigated mechanisms involved in the disruption of TJ integrity by Cd exposure using an in vitro human air-liquid-interface (ALI) airway tissue model derived from normal primary human bronchial epithelial cells.

**Methods:**

ALI cultures were exposed to noncytotoxic doses of CdCl_2_ basolaterally and TJ integrity was measured by Trans-Epithelial Electrical Resistance (TEER) and immunofluorescence staining with TJ markers. PCR array analysis was used to identify genes involved with TJ collapse. To explore the involvement of kinase signaling pathways, cultures were treated with CdCl_2_ in the presence of kinase inhibitors specific for cellular Src or Protein Kinase C (PKC).

**Results:**

Noncytotoxic doses of CdCl_2_ resulted in the collapse of barrier function, as demonstrated by TEER measurements and Zonula occludens-1 (ZO-1) and occludin staining. CdCl_2_ exposure altered the expression of several groups of genes encoding proteins involved in TJ homeostasis. In particular, down-regulation of select junction-interacting proteins suggested that a possible mechanism for Cd toxicity involves disruption of the peripheral junctional complexes implicated in connecting membrane-bound TJ components to the actin cytoskeleton. Inhibition of kinase signaling using inhibitors specific for cellular Src or PKC preserved the integrity of TJs, possibly by preventing occludin tyrosine hyperphosphorylation, rather than reversing the down-regulation of the junction-interacting proteins.

**Conclusions:**

Our findings indicate that acute doses of Cd likely disrupt TJ integrity in human ALI airway cultures both through occludin hyperphosphorylation via kinase activation and by direct disruption of the junction-interacting complex.

## Introduction

Cadmium (Cd) is a highly toxic heavy metal present in environmental pollution and tobacco smoke [1]. For the general population, cigarette smoke is a major source of Cd exposure, with approximately 10-20% of the Cd in mainstream smoke inhaled and 50% of the inhaled dose absorbed via the respiratory system. Thus, smokers usually have significantly higher urine and blood Cd levels than nonsmokers [2,3]. Repeated low doses of Cd, such as those from daily cigarette smoking, accumulate in various organs, leading to pulmonary, cardiovascular, and renal toxicity [4-6].

It is postulated that Cd disturbs tissue homeostasis via multiple mechanisms, including inducing apoptosis and oxidative stress, inhibiting DNA repair, promoting DNA demethylation, interfering with essential metals, and disrupting E-cadherin-mediated cell adhesion [6]. Recent studies conducted in renal tubule epithelial cells, rat seminiferous epithelial cells, and human lung epithelial cells revealed that exposure to Cd also can disrupt intercellular tight junctions (TJs) and that the modes of action for the resulting toxicity likely are tissue-specific [7-10]. Given that pulmonary disease is a common outcome of Cd-related exposures and that the human airway is exposed to Cd through, e.g., cigarette smoke, airway epithelium could be a major target for Cd-mediated TJ disruption. Whether or not such toxicity occurs and the underlying mechanism for this toxicity, therefore, warrants further investigation.

The TJ is a highly dynamic molecular structure involved in maintaining and regulating tissue permeability and polarity [11]. The basic structure of TJs comprises a network of transmembrane proteins and cytoplasmic plaques connected by adaptor proteins [12]. Dysregulation of TJ permeability has been implicated in a variety of pathological conditions in pulmonary tissues, including asthma and lung cancer [13]. Possible molecular targets leading to TJ disruption have been studied extensively. In addition to the well-understood membrane components that are mainly involved in mediating intercellular adhesion, the cytoplasmic fractions of the TJs increasingly have been recognized for their essential roles in regulating TJ biogenesis and transmitting cellular signals between different components of the TJ plaques [12]. It is believed that the expression of TJ-associated proteins as well as their subcellular localization and phosphorylation status are essential in maintaining the barrier function of TJs in response to various stimuli, such as oxidative stress, inflammation, and cytokines [14,15]. The function of phosphorylation modification may involve both modulating interactions between TJ-associated molecules and signal transduction upon internal and external stimulation [16,17]. Several protein kinases and phosphatases have been identified that are directly involved in occludin phosphorylation on multiple residues [18].

Well-differentiated human airway air-liquid-interface (ALI) tissue cultures derived from normal primary human bronchial epithelial (NHBE) cells develop mature TJs and retain all major cell types found in human airway epithelium. Thus, they have emerged as an advanced in vitro tissue model that closely recapitulates in vivo airway epithelial tissue architecture and function. In this study, we explored molecular events underlying Cd-mediated TJ disruption in human airway ALI tissue models.

## Methods

### Reagents

CdCl_2_ was purchased from MP Biomedicals (CAS No. 10108-64-2; purity > 99.0%; Santa Ana, CA). Mouse and rabbit anti-occludin antibodies and rabbit anti-ZO-1 antibody were purchased from Invitrogen (Grand Island, NY). Mouse anti-VAP-33 antibody was obtained from BD Biosciences (San Jose, CA). Rabbit anti-TJAP1 antibody was obtained from Abcam (Cambridge, MA). Mouse anti-GAPDH and rabbit anti-cingulin antibodies were purchased from Sigma-Aldrich (St. Louis, MO). Mouse anti-p-Tyr antibody PY99, c-Src kinase inhibitor I, and pan Protein Kinase C (PKC) inhibitor (GF 109203X) were purchased from Santa Cruz Biotechnology (Dallas, TX). Mouse anti-p63 and rabbit anti-Ki-67 antibodies were obtained from Thermo Scientific (Pittsburgh, PA) and Lab Vision Corporation (Fremont, CA), respectively. Horseradish peroxidase-conjugated goat anti-mouse and goat anti-rabbit antibodies were purchased from Vector Laboratories (Burlingame, CA). Alexa Fluor®488 goat anti-mouse IgG and Alexa Fluor®594 goat anti-rabbit IgG were obtained from Invitrogen. IRDye 680RD goat anti-mouse IgG, IRDye 680RD goat anti-rabbit IgG, and IRDye 800CW goat anti-mouse IgG secondary antibodies were purchased from LI-COR (Lincoln, NE).

### Airway epithelial ALI culture

NHBE cells cryopreserved at Passage 1 were purchased from Lonza (Walkersville, MD). Cells were quickly thawed at 37°C upon receipt and expanded on collagen-coated P100 tissue culture dishes in bronchial epithelium growth medium (BEGM, Lonza). ALI cultures were established using a PneumaCult ALI medium kit and by following the manufacturer’s instructions (STEMCELL Technologies, Vancouver, Canada). Briefly, after the cells reached approximately 70% confluency in the P100 tissue culture dishes, they were induced by incubation for 24 h in Induction Medium provided by the ALI medium kit. The induced cells were collected with 0.025% Trypsin-EDTA, resuspended in Expansion Medium provided by the medium kit, and seeded onto 24-well collagen-coated PET cell culture inserts (BD Biosciences, San Jose, CA). The cells were incubated in Expansion Medium added to both the apical and basolateral chambers of the culture inserts until they reached 100% confluency. The apical medium then was removed and the cells were fed basolaterally only with kit-supplied serum- and bovine pituitary extract-free Maintenance Medium. The basolateral medium was replaced every 2 or 3 days for approximately 4 weeks until the cultures became fully differentiated.

### Histology

ALI cultures were washed briefly with phosphate-buffered saline (PBS, pH 7.4) and fixed in 10% neutral buffered formalin for 48 h. The membranes were excised from the culture inserts with a surgical blade and embedded in paraffin. Five-μm-thick tissue sections were cut, mounted on slides, and deparaffinized by processing through a series of xylene and ethanol solutions. Hematoxylin and eosin (H&E) staining was conducted using a Leica Autostainer. Deparaffinized tissue sections also were stained for p63, Ki-67, and mucus-producing goblet cells. For p63 and Ki-67 staining, antigens were retrieved by boiling the tissue sections in 0.01 M citrate buffer, pH 6.0, for 15 min (3 × 5 min/run) using a microwave oven. After the slides were cooled to room temperature, they were washed in dH_2_O for 5 min. The slides then were treated with 3% hydrogen peroxide containing 1% sodium azide for 10 min to quench endogenous peroxidases. Following blocking the nonspecific binding in 0.5% casein for 20 min, the slides were incubated for 1 h at room temperature with antibodies for p63 or Ki-67 diluted 1:100 and 1:200, respectively, in PBS containing 1% bovine serum albumin (BSA, Sigma-Aldrich). After the conclusion of the primary antibody incubation, the slides were incubated with the respective horseradish peroxidase-conjugated secondary antibodies for 30 min at room temperature. Staining was developed with diaminobenzidine (DAB, Sigma-Aldrich) substrate for 5 min at room temperature. Sections then were counter-stained with hematoxylin and mounted with Permount mounting medium (Thermo Scientific). Mouse IgG, rabbit IgG, or PBS was added in the place of primary antibodies to serve as the negative staining controls.

Mucus-producing goblet cells were identified by periodic acid Schiff’s (PAS) staining. Slides with deparaffinized tissue sections were incubated in periodic acid solution for 5 min at room temperature. The slides then were washed with H_2_O and incubated in Schiff’s reagent for 30 min at room temperature. The slides were washed again in running tap water and counter-stained with hematoxylin.

### Cd exposure conditions

Treatment medium containing various concentrations of CdCl_2_ was prepared by diluting a stock solution in Maintenance Medium. Exposure to CdCl_2_ was carried out from the basolateral side of the ALI cultures to mimic the systemic (blood) exposure of airway epithelium to Cd. Four hundred μL treatment medium were added to the basolateral compartments for 24, 48, or 72 h. After the treatment, the basolateral medium was removed and both the apical and basolateral surfaces of the cultures were washed with PBS once before proceeding to the following analyses.

### Trans-epithelial electrical resistance (TEER)

TEER measurements were made using an Endohm-6 tissue resistance chamber and an epithelial volt-ohmmeter (EVOM2, World Precision Instruments, Sarasota, FL). The EVOM2 was calibrated using a test electrode prior to the measurement. The culture insert was positioned in the center of the Endohm-6 chamber containing 1 mL of the electrolyte solution (0.9% NaCl, 1.25 mM CaCl_2_, 10 mM HEPES); and 300 μL of the electrolyte solution were added to the apical chamber of the culture insert. TEER values were taken 3 times per data point and an average was calculated. An empty culture insert was used to correct for the background resistance. Three cultures were used for each treatment concentration and time point.

### Cell viability

Cell viability was measured using the CellTiter 96® Aqueous Non-radioactive Cell Proliferation MTS Assay (Promega, Madison, WI). The MTS assay is based on the bio-reduction of a tetrazolium compound (MTS) to a soluble formazan product by metabolically active cells. The amount of formazan product formed is directly proportional to the number of live cells, thus providing a quantitative measure of cell viability. After TEER measurement, 200 μL fresh Maintenance Medium containing the MTS/PMS reagent were added to the apical compartment of the culture inserts and incubated for 1 h at 37°C to allow the metabolic conversion of tetrazolium to formazan. One hundred and twenty μL of the apical medium then were transferred to a clear 96-well plate. The optical density of the apical medium was measured at 490 nm using a Synergy H4 plate reader (BioTek, Winooski, VT). Triplicate cultures were used for each treatment concentration and time point.

### Immunofluorescence

Double immunofluorescence labeling with Zonula occludens-1 (ZO-1) and occludin was conducted by following the method described previously [19]. Prior to the treatment, the accumulated mucus was removed by washing the apical side of the ALI cultures thoroughly with PBS. The cultures then were treated for 24 h with various concentrations of CdCl_2_ in the presence or absence of inhibitors of c-Src or PKC. At the end of the treatment, the cultures were washed with PBS twice and permeabilized in methanol for 30 min at −20°C. After washing with PBS for 15 min (3 × 5 min/wash), the cultures were incubated for 1 h with 100 μL of goat anti-rabbit ZO-1 and goat anti-mouse occludin antibodies (both diluted 1:500 in PBS containing 10% normal goat serum), followed by washing with PBS 3 times (5 min/wash) to remove the unbound antibodies. One hundred μL of Alexa Fluor® 488 goat anti-mouse IgG or Alexa Fluor® 594 goat anti-rabbit IgG (both diluted 1:500 in PBS with 10% normal goat serum) were added to the apical chamber and the cultures were incubated in dye-conjugated secondary antibodies for 1 h in the dark. The cultures were again washed 3 times with PBS (5 min/wash) and post-fixed with freshly prepared 4% paraformaldehyde for 30 min at room temperature in the dark. The membranes were excised from the plastic insert support using a surgical blade and mounted on a positively charged microscope slide with 30 μL VECTASHIELD® Mounting Medium containing 4’,6-diamidino-2-phenylindole (DAPI) (Vector Laboratories). Images were captured with a Nikon fluorescence microscope equipped with appropriate filters.

### Human tight junction PCR array

Total RNA was isolated using an miRNeasy Mini Kit (Qiagen, Gaithersburg, MD) by following the manufacturer’s instructions. The quality of the total RNA was assessed using the Bio-Rad Experion Automated Electrophoresis System (Hercules, CA) and RNA samples with an RNA Integrity Number ≥ 9.5 were used for cDNA synthesis. Four hundred ng of the total RNA were used to synthesize cDNAs using a Qiagen RT^2^ First Strand Kit.

Gene expression profiles were generated using human TJ RT^2^ Profiler PCR arrays (SABiosciences, Gaithersburg, MD) and a 7500 Fast Real-Time PCR System (Applied Biosystems, Carlsbad, CA). The vehicle-treated control was conducted in quadruplicate (N = 4) and each CdCl_2_ treatment was performed in triplicate (N = 3). Ct values were normalized using the house-keeping gene, *Hprt1*, prior to data analysis. Gene expression changes were calculated using RT^2^ Profiler PCR Array Data Analysis v3.5 software (available on-line at: http://www.sabiosciences.com/pcrarraydataanalysis.php). Gene expression was compared between the vehicle-treated control and the two CdCl_2_ treatments using the ΔΔCt method. Student’s *t*-test conducted on quadruplicate 2^-ΔΔCt^ values for each gene in the vehicle-treated control and on triplicate values for the CdCl_2_-treated groups was used to assess the significance of differences in gene expression. Genes with fold changes ≥ 1.5 and *p*-values ≤ 0.05 were selected as candidates that respond to Cd exposure.

Principal component analysis (PCA) was conducted using ArrayTrack (U.S. FDA/National Center for Toxicological Research, Jefferson, AR). Briefly, PCR array data corrected for backgrounds were imported into ArrayTrack and normalized using Total Intensity Normalization. Expression ratios (treated/control) were log_2_-transformed. The transformed data were used for PCA to evaluate the effect of Cd exposure. Among the nine principal components considered by the ArrayTrack software, the program selected the first, second and third components as the most informative; these accounted for 45.79, 31.70 and 10.12% of the variance in the correlation matrix, respectively.

### Immunoblotting

Total cell lysates were prepared using the Pierce M-PER Mammalian Protein Extraction Reagent supplemented with 1 × SIGMA*FAST*™ Protease Inhibitor Cocktail. Protein concentrations were determined using the BCA protein assay. Ten μg aliquots of total protein were loaded onto a NuPage® Novex® 4-12% Bis-Tris gel (Invitrogen) and proteins were separated in MES running buffer at 200 V for 35 min. The separated proteins then were transferred to a nitrocellulose membrane at 30 V for 1 h and blocked with Odyssey Blocking Buffer (LI-COR) for 1 h at room temperature with gentle rocking. Subsequently, the membrane was incubated overnight at 4°C with antibodies diluted in Odyssey Blocking Buffer with 0.1% Tween-20. Proteins were visualized with IRDye-conjugated secondary antibodies and a LI-COR Odyssey CLx Imaging System.

### Co-immunoprecipitation

Co-immunoprecipitation (co-IP) was conducted using the Pierce Co-IP kit according to the manufacturer’s instructions. Briefly, 40 μg of rabbit anti-occludin antibody were immobilized with AminoLink Plus Coupling Resin and stored at 4°C until the analysis. Cells were lysed in 100 μL IP Lysis/Wash Buffer supplemented with 1 × SIGMA*FAST*™ Protease Inhibitor Cocktail. Protein concentrations were determined using the BCA protein assay and 500 μg of total protein were precleared through 40 μL of Control Agarose Resin to minimize nonspecific protein binding. Equal volumes of the precleared cell lysate were added to the antibody-coupled resin and incubated overnight at 4°C with gentle mixing. The protein complex was eluted in 35 μL Elution Buffer and 20 μL of the elute were analyzed for p-Tyr by immunoblotting.

### Statistical analysis

All data are presented as mathematical mean values ± standard error of the mean. Differences between the vehicle-treated control and CdCl_2_-treated groups were assessed by using one-way ANOVA followed by Dunnett’s test provided by the SigmaPlot version 11 statistics package. Data were tested for normality. *p*-values ≤ 0.05 were considered statistically significant.

## Results

### Morphological evaluation of the ALI tissue model

**Figure 1:**
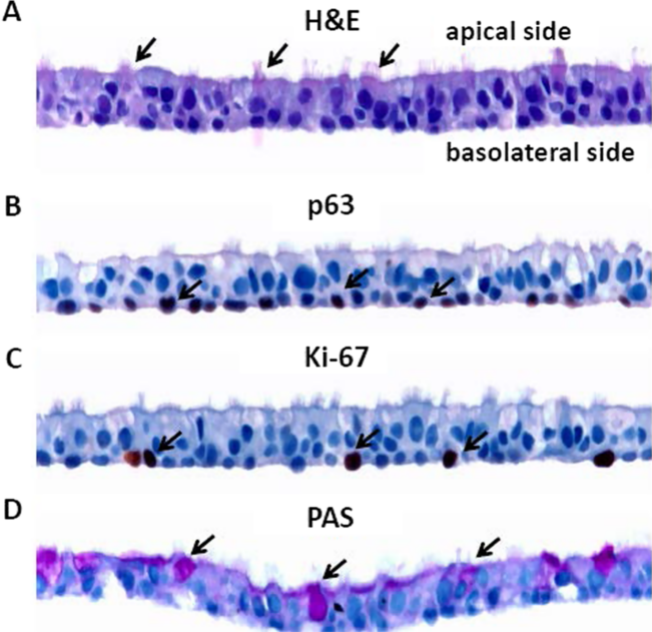
**Morphological characterization of ALI human airway cultures.** ALI cultures were fixed in 10% formalin 5 weeks after the initial seeding onto the permeable membrane support. Tissue sections were stained with H&E **(A)**, p63 **(B)**, Ki67 **(C)**, and PAS **(D)**. Examples of positively stained cells are indicated by arrows. The arrows in **(A)**, **(B)**, **(C)**, and **(D)** indicate ciliated cells, basal cells, actively proliferating cells, and goblet cells, respectively.

The morphology of differentiated ALI cultures was evaluated by histological methods 5 weeks after the initial seeding onto the permeable membrane support. H&E staining of the paraffin-embedded tissue sections indicated that the ALI cultures were fully differentiated into a pseudostratified mucociliary epithelium, with cells resembling goblet cells interspersed among ciliated cells along the apical side of the cultures (Figure 1A). Different types of epithelial cells were further distinguished by cell-specific markers. The cuboidal-shaped basal cells were identified by staining with the progenitor cell marker, p63 (Figure 1B). Cells expressing p63 were found exclusively along the basolateral side of the ALI culture. Cells undergoing active proliferation also were located only on the basolateral side, as demonstrated by staining with the cellular proliferation marker, Ki-67 (Figure 1C). PAS staining, which detects the glycoprotein and glycolipid components in mucin, was used to identify the mucus-secreting goblet cells (Figure 1D). Consistent with the H&E staining, PAS-positive goblet cells were distributed along the apical side of the culture. Residual secreted mucus also was detected on the apical side by PAS staining.

### CdCl_2_ reduces TEER values and induces cellular toxicity

**Figure 2:**
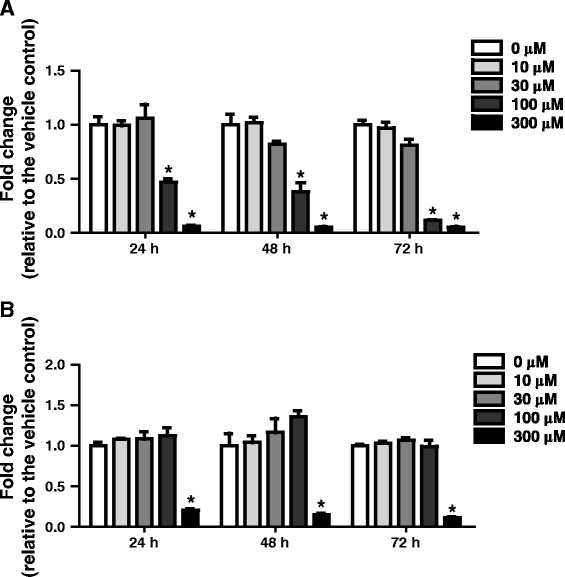
**Evaluation of transepithelial electrical resistance (TEER) and cellular toxicity in CdCl**_**2**_ **-treated human ALI cultures.** Cultures were treated from the basolateral side with various concentrations of CdCl_2_ for 24, 48, or 72 h. The cultures were washed briefly in PBS and TEER measurements **(A)** were conducted before processing the cultures for cytotoxicity evaluation using the MTS assay **(B)**. Data (N = 3) are presented as means ± standard deviation. *p < 0.05 was considered to be significant compared to the vehicle-treated control.

TEER measurements were made on CdCl_2_-treated ALI cultures, followed by the evaluation of cytotoxicity with the MTS assay. Cultures were treated basolaterally with various concentrations of CdCl_2_ for 24, 48, or 72 h as described in the Methods section. CdCl_2_ caused dose- and time-dependent decreases in TEER values. Treatment with 100 μM CdCl_2_ produced an approximate 50% reduction in TEER following both the 24- and 48-h exposures (Figure 2A), and increasing the treatment duration to 72 h further decreased the TEER values close to background levels (i.e., the resistance of an empty culture insert). Treatments with 10 and 30 μM CdCl_2_ had only minor effects on TEER values, whereas the integrity of the cultures was completely disrupted with a dose of 300 μM CdCl_2_ at all sampling times.

The MTS assay revealed a trend in dose–response toxicity similar to that observed with the TEER measurements (Figure 2B). Doses up to 100 μM CdCl_2_ did not significantly decrease cell viability at any of the time points tested. A dose of 300 μM, however, completely inhibited the metabolic activities of the cultures at all sampling time points, indicating that the cultures were no longer viable. The IC_50_ value for CdCl_2_ determined by the MTS assay fell between 100 μM and 300 μM, which was higher than that of approximately 100 μM estimated by TEER measurements after the 24-h treatment. Given that the 100 μM CdCl_2_ was noncytotoxic and induced an approximately 50% reduction in TEER measurement, we selected concentrations of 30, 50, and 100 μM for our subsequent mechanism studies.

### Acute treatment with CdCl_2_ disrupts TJ integrity

**Figure 3:**
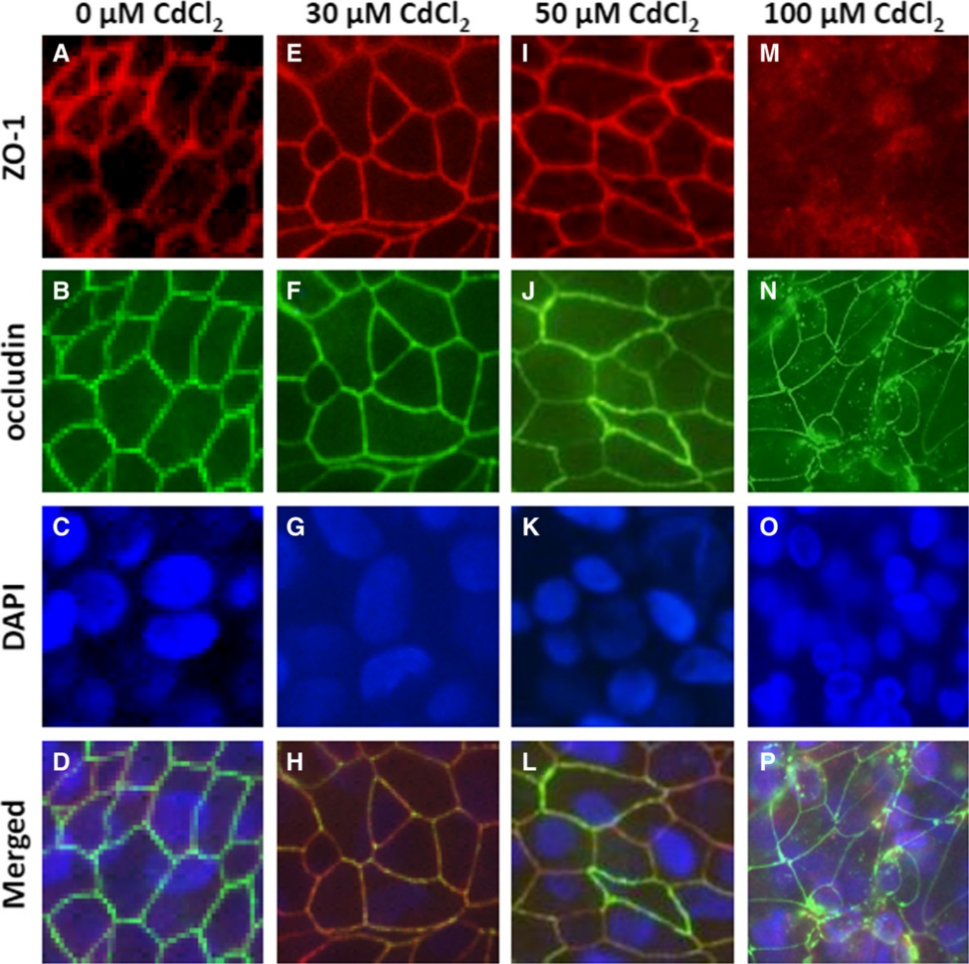
**Induction of TJ disruption by CdCl**_**2**_ **.** Cultures were treated from the basolateral side with 30, 50 and 100 μM CdCl_2_ for 24 h, and immunofluorescence staining was performed with antibodies for ZO-1 and occludin. Merged images demonstrated the colocalization of ZO-1 and occludin on the cellular borders in vehicle-treated control and 30 μM- and 50 μM-treated groups. Note apparent condensation of staining of ZO-1 and occludin in 100 μM CdCl_2_-treated cultures. Descriptions of the individual lettered panels are given in the text.

The drop in TEER values indicated a possible disruption of TJ function caused by CdCl_2_ treatment. Thus, alterations in TJ integrity in response to CdCl_2_ exposures were further investigated using immunofluorescence staining of two TJ markers, ZO-1 and occludin. In vehicle-treated control cultures, both ZO-1 and occludin exhibited a well-defined localization around cell borders, indicating the presence of intact TJs (Figure 3, panels *A* and *B*). The merged images of ZO-1 and occludin fluorescence staining further revealed their colocalization at the TJs (Figure 3, panel *D*). Treatments with 30 μM and 50 μM of CdCl_2_ did not alter the TJ integrity and colocalization of ZO-1 and occludin after 24 h (Figure 3, panels *E* and *F* and panels *I* and *J*), an observation consistent with the TEER measurement (Figure 2A). After the 24-h treatment with 100 μM CdCl_2_, however, the staining of these two TJ markers was not limited to cellular boundaries as observed in vehicle-treated control group. Both ZO-1 and occludin exhibited partial membrane delocalization and redistribution to the cytoplasm (Figure 3, panels *M* and *N*). It was noteworthy that CdCl_2_ only partially disrupted TJs at 100 μM, as the colocalization of ZO-1 and occludin at cellular boundaries was still visible in some areas of the cultures.

### Acute treatment with CdCl_2_ modulates genes associated with TJs

The human TJ RT^2^ Profiler PCR array was used to measure the relative expression of 84 genes involved in TJ assembly and regulation. Cultures were treated basolaterally with vehicle, 30 μM and 100 μM CdCl_2_ for 24 h. The selection of the doses was based upon the extent of TJ disruption elicited by these doses inferred from the TEER measurements and staining for ZO-1 and occludin.

**Figure 4:**
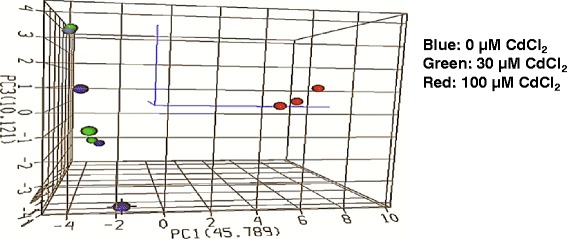
**3D depiction of principal component analysis (PCA) of gene expression in cultures treated with vehicle, 30 μM CdCl**_**2**_ **, or 100 μM CdCl**_**2**_ **.** Sixty five genes that met the criteria defined by the RT^2^ Profiler PCR Array Data Analysis v3.5 software from SABiosciences were uploaded to the ArrayTrack program. The PCA is based on the log_2_ ratios and expression profiles across all the 65 genes in the PCR array. The blue, green, and red dots indicate cultures treated with vehicle, 30 μM CdCl_2_, and 100 μM CdCl_2_, respectively. The first three principal components are plotted. The captured variance of PC1 (first principal component), PC2 (second principal component; the label is not shown), and PC3 (third principal component) were 45.79%, 31.70%, and 10.12%, respectively.

Nineteen genes had average threshold cycles greater than 30 and thus were excluded from the subsequent analysis. Principal component analysis (PCA) was performed to investigate the relationships for gene expression among the samples from the different dose groups (Figure 4). The vehicle-treated control samples and the low dose samples were loosely grouped together, indicating a minimum effect on gene expression associated with the 30 μM CdCl_2_ treatment. Samples treated with 100 μM CdCl_2_ were more tightly grouped together and separated from both the vehicle-treated control and low dose groups. Principal component 1 was found to be a convenient parameter to differentiate the treatment effects elicited by different doses of CdCl_2_.

Using the criteria defined in our study, 3 genes were modulated by 30 μM CdCl_2_ and 29 genes exhibited significant changes in their expression ratios (treatment/control) in response to the 100 μM CdCl_2_ treatment (Table 1). Among the 29 genes that demonstrated marked changes to 100 μM CdCl_2_, 11 genes were up-regulated and 18 genes were down-regulated. Notably, claudin 2 displayed differential responses to the 30 μM- and 100 μM-treatments.

**Table 1:** **Significantly regulated TJ-related genes in response to CdCl**_**2**_ **treatment in human airway epithelial ALI cultures (fold change ≥ 1.5,** ***p*** **value < 0.05)**

**Gene name**	**Gene description**	**Biological function**	**Group 1 (30 μM)**		**Group 2 (100 μM)**	
			**Fold change**	***p*** **value**	**Fold change**	***p*** **value**
**Up-regulated**						
ICAM1	Intercellular adhesion molecule 1	Cell surface receptors			13.89	0.000114
CRB3	Crumbs homolog 3 (Drosophila)	Cell surface receptors			3.95	0.000507
CLDN1	Claudin 1	Cell surface receptors			2.99	0.000232
CLDN4	Claudin 4	Cell surface receptors			2.57	0.012412
OCLN	Occludin	Cell surface receptors			2.53	0.000418
ARHGEF2	Rho/rac guanine nucleotide exchange factor 2	G Protein signaling			2.15	0.007405
CLDN2	Claudin 2	Cell surface receptors			2.14	0.045285
TJP1	Tight junction protein 1 (Zona occludens-1)	Junction interacting proteins			2.13	0.00451
SMURF1	SMAD specific E3 ubiquitin protein ligase 1	Cytoskeleton Regulator/G Protein Signaling			1.71	0.005857
CLDN12	Claudin 12	Cell surface receptors			1.65	0.008749
JAM3	Junctional adhesion molecule 3	Junction interacting proteins			1.62	0.015438
AMOTL1	Angiomotin like 1	Junction interacting proteins/Cytoskeleton regulation	1.99	0.009748		
HCLS1	Hematopoietic cell-specific Lyn substrate 1	Junction interacting proteins	1.62	0.017181		
**Down-regulated**						
MPDZ	Multiple PDZ domain protein	Junction interacting proteins			−7.35	0.000001
PTEN	Phosphatase and tensin homolog	Protein kinase signaling			−4.22	0
ICAM2	Intercellular adhesion molecule 2	Cell surface receptors			−4.01	0.019093
CGN	Cingulin	Junction interacting proteins			−3.74	0.007739
CDK4	Cyclin-dependent kinase 4	G Protein signaling			−3.58	0.012422
CLDN16	Claudin 16	Cell surface receptors			−3.15	0.000298
MAGI1	Membrane associated guanylate kinase, WW and PDZ domain containing 1	Junction interacting proteins/Protein kinase signaling			−3.13	0.000032
CLDN8	Claudin 8	Cell surface receptors			−2.77	0.000306
CLDN2	Claudin 2	Cell surface receptors	−1.96	0.03226		
GNAI1	Guanine nucleotide binding protein (G protein), alpha inhibiting activity polypeptide 1	G Protein signaling			−2.70	0.000373
VAPA	VAMP (vesicle-associated membrane protein)-associated protein A, 33kDa	Junction interacting proteins			−2.20	0.000723
TJAP1	Tight junction associated protein 1 (peripheral)	Junction interacting proteins			−2.20	0.028018
ASH1L	Ash1 (absent, small, or homeotic)-like (Drosophila)	Cytoskeleton Regulator			−2.14	0.000877
SYMPK	Symplekin	Junction interacting proteins			−2.07	0.040784
CSNK2A2	Casein kinase 2, alpha prime polypeptide	Protein kinase signaling			−1.97	0.004506
CSDA	Cold shock domain protein A	Junction interacting proteins/Cytoskeleton regulator			−1.73	0.000039
MPP6	Membrane protein, palmitoylated 6 (MAGUK p55 subfamily member 6)	Protein kinase signaling			−1.71	0.0007
MLLT4	Myeloid/lymphoid or mixed-lineage leukemia (trithorax homolog, Drosophila); translocated to, 4	Junction interacting proteins			−1.60	0.014185
CSNK2A1	Casein kinase 2, alpha 1 polypeptide	Protein kinase signaling			−1.55	0.002999

**Figure 5:**
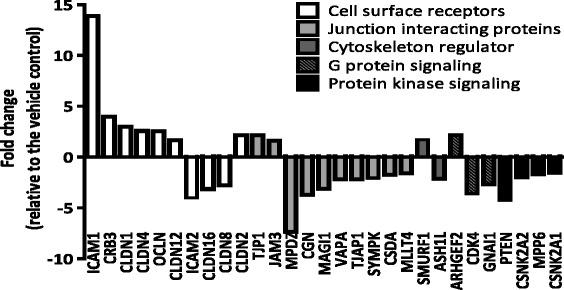
**Expression changes of TJ-related genes in cultures exposed basolaterally to 100 μM CdCl**_**2**_ **.** The means of the gene expression changes are plotted. All genes presented here have a fold change ≥ 1.5 and *p*-value < 0.05. N = 4 for vehicle-treated control group; N = 3 for 100 μM CdCl_2_-treated group.

Genes that were differentially expressed are involved in a wide range of TJ-related biological processes, including cell surface receptors, the junction-interacting complex, G protein signaling, and protein kinase signaling (Figure 5). Nearly 40% of the genes differentially expressed by 100 μM CdCl_2_ were involved with the functions of the cytoplasmic junctional-protein complex. Cell surface receptors and protein kinase signaling molecules also were among the top groups of genes that were significantly modulated by Cd exposure.

**Figure 6:**
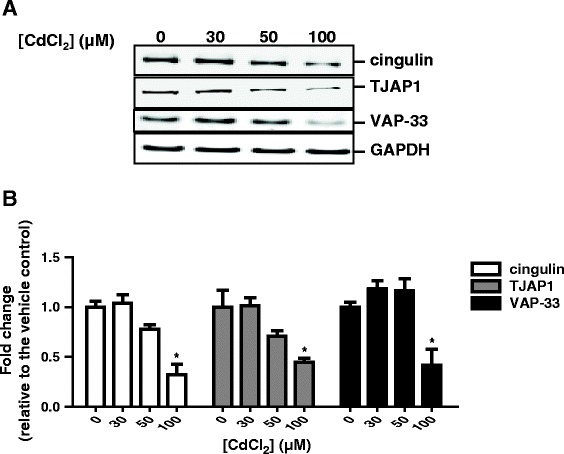
**Cingulin, TJAP1, and VAP-33 protein expression changes in response to CdCl**_**2**_ **treatment.** Cultures were treated from the basolateral side with various concentrations of CdCl_2_ for 24 h, and whole cell lysate was collected and separated by electrophoresis. The expression of cingulin, TJAP1, and VAP-33 was detected by immunoblotting using specific antibodies. **(A)**. Representative Western blots. **(B)**. Density of the bands were quantified and statistically analyzed (N = 3). *Indicates p < 0.05 compared to the vehicle-treated control.

Since genes involved in the junctional-protein complex were among the top gene groups modulated by 100 μM CdCl_2_, three differentially expressed junctional-interacting genes, e.g., cingulin, tight junction interaction protein 1 (TJAP1), and VAP-33, were selected and changes in their protein expression were further examined by immunoblotting. Cingulin and TJAP1 exhibited clear dose-dependent decreases in their protein expression with increasing concentrations of CdCl_2_; the expression of VAP-33 also was decreased but its decrease was less dose-responsive (Figure 6A). Quantitative assessment, however, indicated that treatment with 100 μM CdCl_2_ decreased the expression of all three junctional-interacting proteins by approximately 50%, decreases that were statistically significant (Figure 6B).

### Inhibitors of c-Src and PKC preserve TJ integrity by inhibiting occludin Tyr phosphorylation, rather than reversing the down-regulation of junctional interacting proteins

**Figure 7:**
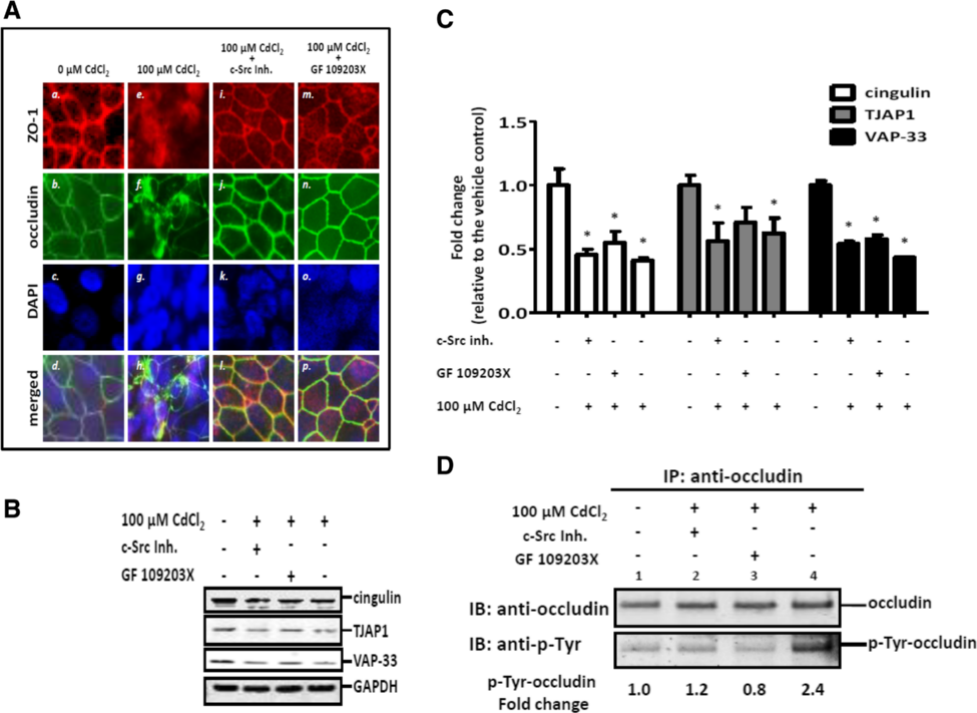
**Protective effects of kinase inhibitors for c-Src and PKC on Cd-induced TJ disruption. (A)**. TJ integrity was assessed using immunofluorescence staining of ZO-1 and occludin. Cotreatment of CdCl_2_ and kinase inhibitors prevented Cd-induced TJ disruption. Descriptions of the individual lettered panels are given in the text. **(B)**. Representative Western blots showing protein expression of cingulin, TJAP1, and VAP-33. Kinase inhibitors failed to prevent the down-regulation of these junction-interacting proteins. **(C)**. Density of the Western blots in Figure 7B. were quantified and statistically analyzed (N = 3). *Indicates p < 0.05 compared to the vehicle treated control. **(D)**. Tyr-phosphorylation of occludin was modulated by CdCl_2_. Cultures were treated from the basolateral side with 100 μM CdCl_2_ in the presence or the absence of kinase inhibitors for c-Src or PKC. Tyrosine phosphorylated occludin was detected in occludin-enriched immunoprecipitates. Both kinase inhibitors prevented Tyr hyperphosphorylation of occludin.

We postulated that kinase activation might be involved in Cd-mediated TJ disruption. To test our hypothesis, ALI cultures were treated with 100 μM CdCl_2_ in the presence or absence of inhibitors of c-Src or PKC. The biological consequences of kinase inhibition on TJ integrity were examined by immunofluorescence staining of ZO-1 and occludin. Treatment of ALI cultures with either kinase inhibitor alone did not change the staining patterns of ZO-1 and occludin, both of which were localized on cellular borders (not shown) as observed in vehicle-treated control cultures (Figure 7A, panels *a* and *b*). Consistent with our initial observations, 100 μM CdCl_2_ partially disrupted TJ integrity (Figure 7A, panels *e* through *h*). Concurrent treatment with CdCl_2_ and the kinase inhibitors, however, effectively preserved the intact TJ structures, as demonstrated by the well-defined staining for ZO-1 and occludin and their colocalization along the cellular borders (Figure 7A, panels *i* through *p*).

The effects of kinase inhibition on the protein expression of the select junctional-interacting proteins were further explored by immunoblotting. Cotreatment with either of the kinase inhibitors did not prevent the CdCl_2_-induced down-regulation of these proteins (Figure 7B). Approximate 50% decreases in the expression of cingulin and VAP-33 (*p* < 0.05) were observed in all treated groups compared to the control; while the expression of TJAP1 also was decreased in all treated groups, cotreatment with CdCl_2_ and the PKC inhibitor failed to significantly down-regulate its expression (Figure 7C).

Since the protective effect of the kinase inhibitors on TJ disruption did not appear to involve the junctional-interacting proteins, we postulated that Cd exposure might alter the phosphorylation status of occludin on Tyr residues, and consequently cause TJ collapse. Because of the lack of an antibody specifically recognizing p-Tyr-occludin, occludin was first enriched by immunoprecipitation of equal amounts of whole cell lysate and Tyr-phosphorylated occludin was detected in the eluate using an antibody raised against Tyr-phosphorylated proteins. The level of total occludin was similar in all treatment groups (Figure 7D, upper panel). Treatment with CdCl_2_ increased occludin Tyr phosphorylation by approximately 2.5-fold (Figure 7D, lower panel, lane 4 vs. lane 1). Concurrent treatment with CdCl_2_ and inhibitors for c-Src or PKC effectively prevented the increase in occludin phosphorylation (Figure 7D, lower panel, lanes 2 and 3 vs. lane 4).

## Discussion

In this study, we investigated the effects of Cd on the integrity of TJs formed in an in vitro airway ALI tissue model derived from primary NHBE cells. Cd was selected as a test compound because of its reported disruption of TJs formed by many cell types [7-10] and its potential for airway exposure due to its presence in cigarette smoke [20]. Exposure of respiratory epithelium can occur by two routes, directly to the luminal (air interface) side of the airway through exposure to Cd in aerosols (e.g., cigarette smoke) or by systemic exposure to Cd circulating in the blood. In our study we exposed the ALI cultures from the basolateral side by adding Cd to the basal medium. This exposure mimics a biologically relevant route of exposure (i.e., systemic exposure), but also was done for a practical reason. Apical exposure of ALI culture would ideally use an aerosol of the test agent delivered in appropriately designed exposure chambers. Exposure of ALI cultures to aqueous solutions of Cd (e.g., dissolved in a small volume of PBS or H_2_O) from the apical side is possible, albeit less of a mimic of in vivo respiratory exposure. However, we found that the aqueous vehicle temporarily affects TJ integrity. We observed that a 24-h treatment at the air-liquid interface with only PBS significantly decreased TEER values, an effect that was reversible several hours after removal of the PBS. Basolateral exposure avoids this temporary and possibly confounding factor that apparently results when the apical side of ALI cultures is covered. Apical exposures, using appropriate exposure systems, would provide additional information to inform a more complete accounting of Cd toxicity to airway ALI cultures.

The results of our study confirmed that the structure of the in vitro ALI cultures closely resembles the tissue architecture of in vivo airway epithelium. We further demonstrated that noncytotoxic doses of CdCl_2_ (measured by the MTS assay) compromised TJ barrier function likely via two independent mechanisms, i.e., by up-regulating occludin Tyr phosphorylation via kinase activation and by directly disrupting the junctional-interacting complex crucial for TJ biogenesis and signaling.

The reported blood levels of Cd in smokers [2] are much lower than the doses of CdCl_2_ that produced significant changes in TJ integrity in the ALI cultures. It should be noted that the half-life of Cd is greater than 10 years in humans, and Cd is known to bind to cellular macromolecules and accumulate in cells [2]. Therefore, acute exposure of the ALI cultures may greatly underestimate the effects of chronic exposure to low doses of Cd. We hypothesize that TJ disruption by more physiologically relevant concentrations of Cd may require longer treatment durations, allowing the accumulation of Cd and manifestation of its toxic effects following repeated exposures. Observations made from our acute-treatment study provide insights into the possible mechanisms of Cd toxicity; however, they cannot exclude the possibility that TJ disruption occurs following longer treatment durations with low doses of Cd. Although longer treatments were not attempted in this study, one of the advantages of the airway ALI tissue model is that it maintains its functional and structural properties from weeks to months so that longer term exposures conducted in such models are possible. Note also that the airway models used in our study were prepared from cells derived from the large airways of human lung tissue. ALI cultures also can be prepared using primary cells from human small airway and alveolar tissue and these should be investigated for TJ disruption by Cd in future studies.

Human TJ PCR array and western blotting revealed the dysregulation of the junctional-interacting complex-associated proteins as a possible mechanism for Cd-mediated TJ disruption. The junctional-interacting complex is a multi-molecular structure located in the cytoplasm and involved in bridging membranous TJ components with the actin cytoskeleton. VAP-33 has been implicated in the early recruitment of occludin to TJs and alteration in its expression results in dislocation of occludin from TJ patches [17]. Cingulin is found to connect TJ proteins with actin cytoskeletons through its head sequence and transmit signals during TJ biogenesis and regulation [21-23]. TJAP1 is incorporated at late stages of TJ assembly, and thus is postulated to have a possible role in stabilizing TJ plaques [24]. Considering their multiple functions at various stages of TJ homeostasis, the Cd-induced dysregulation of the junctional interacting proteins observed in our study could have taken place in a staged manner, with down-regulation of VAP-33 occurring after TJAP1 and cingulin, resulting in several possible downstream effects, such as disassembly of the protein-junctional complex, disruption of the actin cytoskeleton, dysregulation of TJ signal transduction, and increased barrier permeability due to redistribution of TJ proteins, eventually leading to the collapse of TJs. In fact, previous reports have demonstrated that Cd-mediated junction interruption is accompanied by disruption of the actin-based cytoskeleton in other in vitro cell model systems [25,26]. Thus, our observations, in conjunction with these previous studies, support a possible signaling cascade triggered during Cd-induced TJ disruption involving dysregulation of both the actin cytoskeleton and junctional-interacting proteins that function as cytoskeletal adaptor proteins in pulmonary airway epithelium.

Consistent with the report of Dokladney et al. [27], our study also indicates that the protein expression of occludin is not affected by Cd exposure, at least at the concentrations tested. Rather, our observations suggest a role for c-Src and PKC in Cd-mediated post-translational modification of occludin in human airway epithelial ALI cultures. Co-administration of c-Src or PKC inhibitors prevented Cd-induced occludin Tyr hyperphosphorylation and the collapse of TJs, suggesting that kinase activation may be an early cellular event triggered by Cd exposure. This effect of Cd on protein kinase activity is not unprecedented; Cd induces conformational changes and modulates the enzymatic activities of protein kinases by either directly binding to their metal-binding dicysteine-containing motifs or substituting for essential metals, such as Zn^2+^, in their regulatory domains [28,29]. For kinases that are involved in TJ maintenance and regulation, modulation of their activities by Cd may elicit a cascade of related down-stream signaling, resulting in the collapse of TJ integrity. For instance, occludin Tyr hyperphosphorylation by kinase activation may attenuate its interaction with other TJ components, leading to its subcellular dislocalization and ultimately, weakening of the TJ structures. It is noteworthy that kinase signaling pathways become dysregulated in several smoking-related pulmonary diseases, such as in non-small cell lung cancer, asthma, chronic obstructive pulmonary disease, and idiopathic pulmonary fibrosis [30]. Thus, modulations of various kinase activities by Cd in these disease settings warrant further investigation.

Several protein kinases modulate TJ barrier functions through occludin phosphorylation. c-Src directly binds to the C-terminal domain of occludin, and Tyr residues Y398/Y402 were identified as the c-Src phosphosites [31,32]. Activation of c-Src by oxidative stress weakens the barrier properties of Caco-2 cells, and is accompanied by the redistribution of occludin and the TJ-associated protein ZO-1 from cellular borders to the cytoplasm [33]. Other protein kinases, such as PKC, also are implicated in phosphorylating occludin and modulating TJ permeability. However, the exact role of the PKC family in occludin phosphorylation and TJ regulation is less clear. Different isoforms of PKCs may regulate TJ integrity by exerting inhibitory or stimulatory effects on occludin phosphorylation in different cellular systems and under different conditions [18]. Even though they mainly catalyze the phosphorylation modifications on Thr and Ser residues, the possible involvement of PKCs in Tyr phosphorylation revealed by our study is consistent with a study conducted on rat hippocampal slices demonstrating direct Tyr phosphorylation of the N-methyl-D-aspartate receptor by PKC activation [34]. Alternatively, it also is possible that Ser and Thr phosphorylation of occludin by PKC activation renders the Tyr residues more susceptible to phosphorylation by c-Src activation, thus suggesting an indirect involvement of PKC in occludin Tyr phosphorylation in response to Cd exposure.

## Conclusions

Our studies suggest a plausible mechanism for Cd toxicity in human airway ALI models. Given the protective effect of kinase inhibitors on TJ integrity, kinase activation is believed to be an early up-stream cellular event triggered by Cd exposure. Furthermore, Tyr hyperphosphorylation of occludin by kinase activation could potentially attenuate occludin interactions with other junctional-interacting proteins and lead to its dislocation from the cytoplasmic membrane. Based on the fact that the kinase inhibitors did not reverse Cd-induced down-regulation of the select junctional-interacting proteins, we conclude that Cd disrupts TJ integrity through at least two independent mechanisms. Besides causing kinase activation and subsequently downstream occludin Tyr hyperphosphorylation, Cd also may directly disrupt the junction interacting complex by inhibiting gene expression for junctional interacting proteins independent of kinase signaling. The direct inhibition of junctional-interacting protein genes may occur through binding of Cd to the promoter region of their DNA. We hypothesize that these combined effects alter the microenvironment of TJ plaques and contribute to compromising the TJ barrier. We, however, view our observations as a starting point for understanding the toxicity of Cd in airway tissue. Further studies should be conducted to explore the effects of apical exposure of the airway tissue models to aerosolized Cd using systems that mimic in vivo exposures, and also to evaluate the effects of chronic apical and basolateral exposures to low doses of Cd. It also is possible that Cd affects TJ integrity differently in different parts of the airway, and studies similar to ours should be conducted using ALI cultures prepared from small airway and alveolar cells.

## Abbreviations

Cd: Cadmium
TJ: Tight junction
ALI: Air-liquid-interface
TEER: Trans-epithelial electrical resistance
PKC: Protein kinase C
ZO-1: Zonula occludens-1
NHBE: Normal primary human bronchial epithelial
TJAP1: Tight junction-associated protein 1
BEGM: Bronchial epithelium growth medium
PBS: Phosphate-buffered saline
H&E: Hematoxylin and eosin
BSA: Bovine serum albumin
DAB: Diaminobenzidine
PAS: Periodic acid-Schiff
EVOM2: Epithelial volt-ohmmeter 2
DAPI: 4’,6-diamidino-2-phenylindole
MTS: A tetrazolium compound
PCA: Principal component analysis
co-IP: Co-immunoprecipitation
BCA: Bicinchoninic acid
ANOVA: Analysis of variance
